# Providing Home-Based HIV Testing and Counseling for Transgender Youth (Project Moxie): Protocol for a Pilot Randomized Controlled Trial

**DOI:** 10.2196/resprot.8562

**Published:** 2017-11-28

**Authors:** Rob Stephenson, Nicholas Metheny, Akshay Sharma, Stephen Sullivan, Erin Riley

**Affiliations:** ^1^ School of Nursing University of Michigan Ann Arbor, MI United States; ^2^ Center for Sexuality and Health Disparities University of Michigan Ann Arbor, MI United States

**Keywords:** transgender persons, HIV, telemedicine

## Abstract

**Background:**

Transgender and gender nonconforming people experience some of the highest human immunodeficiency virus (HIV) rates in the United States, and experience many structural and behavioral barriers that may limit their engagement in HIV testing, prevention, and care. Evidence suggests that transgender and gender nonconforming youth (TY) are especially vulnerable to acquiring HIV, yet there is little research on TY and few services are targeted towards HIV testing, prevention, and care for this population. Telehealth presents an opportunity to mitigate some structural barriers that TY experience in accessing HIV testing, allowing TY to engage in HIV testing and counseling in a safe and nonjudgmental space of their choosing. Project Moxie is an HIV prevention intervention that pairs the use of HIV self-testing with remote video-based counseling and support from a trained, gender-affirming counselor. This study aims to offer a more positive HIV testing and counseling experience, with the goal of improving HIV testing frequency.

**Objective:**

Project Moxie involves a pilot randomized controlled trial (RCT) of 200 TY aged 15-24 years, who are randomized on a 1:1 basis to control or intervention arms. The aim is to examine whether the addition of counseling provided via telehealth, coupled with home-based HIV testing, can create gains in routine HIV testing among TY over a six-month follow-up period.

**Methods:**

This study implements a prospective pilot RCT of 200 TY recruited online. Participants in the control arm will receive one HIV self-testing kit and will be asked to report their results via the study’s website. Participants in the experimental arm will receive one HIV self-testing kit and will test with a remotely-located counselor during a prescheduled video-counseling session. Participants are assessed at baseline, and at three and six months posttesting.

**Results:**

Project Moxie was launched in June 2017 and recruitment is ongoing. As of August 21, 2017, the study had enrolled 130 eligible participants.

**Conclusions:**

Combining home-based HIV testing and video-based counseling allows TY, an often stigmatized and marginalized population, to test for HIV in a safe and nonjudgmental setting of their choosing. This approach creates an opportunity to reduce the high rate of HIV among TY through engagement in care, support, and linkage to the HIV treatment cascade for those who test positive.

**Trial Registration:**

ClinicalTrials.gov NCT03185975; https://clinicaltrials.gov/ct2/show/NCT03185975 (Archived by WebCite at http://www.webcitation.org/6vIjHJ93s)

## Introduction

Evidence increasingly suggests a high prevalence of human immunodeficiency virus (HIV) infections among transgender and gender nonconforming individuals in the United States [1]. Recent studies, drawn largely from samples of transgender women, suggest the prevalence of HIV in this population is at least as high as that experienced by gay, bisexual, and other men who have sex with men (MSM) [2-4]. A 2013 report found that the estimated percentage of transgender women living with HIV in the United States was 22% [4], and among the 3.3 million HIV testing events reported to the Centers for Disease Control and Prevention (CDC) in 2013, the percentage of transgender people who received a new HIV diagnosis was more than three times the national average [1]. Transgender women of color and those under 25 years of age are disproportionately affected by the HIV epidemic [5,6].

Much of what is known about HIV risk behaviors among transgender and gender nonconforming people has been derived from samples of transgender women [1]. The high rate of HIV in this population has been associated with a high prevalence of substance abuse, commercial sex work, condomless anal intercourse, and a lack of knowledge regarding HIV transmission [5,7-10]. While data on other transgender and gender nonconforming populations is sorely lacking, many of the risk factors for HIV seen in transgender women are shared by other groups of transgender and gender nonconforming people, and the prevalence of HIV is thought to be similar across transgender and gender nonconforming populations [3]. While transgender and gender nonconforming youth (TY) are even more underrepresented in the literature, available research from a sample of trans-feminine youth shows that this population experiences multiple forms of discrimination and harassment, significantly increasing the risk of HIV transmission [11,12]. A study of 51 ethnic-minority, female-identifying TY showed higher rates of HIV compared to other racial/ethnic groups, with 41% experiencing difficulty accessing health care, 59% reporting engaging in transactional sex, and 63% reporting difficulty finding employment [9].

Telehealth uses short message service (SMS) messaging, teleconferencing and videoconferencing platforms, social media, and smartphone apps compliant with the Health Information Portability and Accountability Act (HIPAA) to increase access to health care [13]. Given the multiple barriers to accessing HIV prevention and care experienced by transgender and gender nonconforming people, telehealth may be a useful platform for delivering services directly to these populations. Due to high rates of technology use among youth [14], telehealth may be especially useful for addressing the discrimination and harassment TY encounter while attempting to access health care resources [15,16]. Telehealth may also provide an entry point to the health care system in places where no gender-affirming providers or services exist.

Telehealth has already been adapted to provide HIV care services to MSM residing in areas where stigma and minimal lesbian, gay, bisexual, transgender (LGBT)-friendly health care providers exist [17]. SMS-based telehealth efforts have also been implemented to connect MSM in metropolitan Kansas City with an HIV testing counselor to increase the dissemination of evidence-based HIV/sexually transmitted infection (STI) prevention information [18]. Telehealth-based interventions have also been used among rural HIV-positive veterans in the United States, wherein they participated in private teleconferences with HIV specialists [19]. This intervention was shown to increase the veterans’ perceptions of the quality of care provided, and was considered a step towards reducing HIV risk behaviors such as condomless intercourse and multiple sexual partners [19]. While telehealth-based interventions may reduce the stigma associated with in-person appointments by allowing rural MSM and HIV-positive veterans to access services in a place where they feel comfortable, research has not focused on the use of telehealth to provide HIV prevention resources to other marginalized populations, such as TY.

Another important limitation of the current telehealth literature concerns its use as an information dissemination and case management tool. Few studies use this technology for actual clinical care, such as HIV testing. With approval by the US Food and Drug Administration in 2011, high quality (sensitivity 93.64%, 95% CI 82.46-98.66; specificity 99.87%, 95% CI 99.28-100.00 [20]), home-based HIV testing is now available commercially in all 50 states [21]. However, critiques of home-based HIV testing include difficulty interpreting the results, a lack of posttest counseling for the adoption of safer HIV prevention methods, and a lack of proper linkage to care [22]. Adding online counseling through video-chat software to home-based HIV testing has the potential to mitigate these concerns. Through telehealth-delivered counseling, individuals can receive tailored, convenient, and confidential support that due to stigma, discrimination, and/or lack of locally-available services, may not otherwise be received. While there are few telehealth-based HIV prevention interventions published in the literature, results from prior studies show increased knowledge, self-efficacy, and motivation towards effective HIV prevention methods [23-26].

This paper describes the protocol for the first study to combine telehealth and home-based HIV testing for comprehensive, gender-affirming HIV testing and counseling for TY. In this study, approximately 200 TY aged 15-24 years will be randomized into either a control group that receives an HIV self-testing kit or an intervention group that receives an HIV self-testing kit as well as a video-chat counseling session provided by a trained HIV counselor. The main outcome of this study is the frequency of HIV testing in the six months following initial testing. The study aims to examine whether the receipt of an HIV self-testing kit plus a video-chat-delivered counseling session can create changes in HIV testing behavior, sexual risk-behaviors, and linkage to care for newly-diagnosed HIV-positive TY. If successful, this low-cost intervention has the potential to shape the delivery of HIV prevention and care services to this population, who are currently overlooked in HIV programming, research, and prevention efforts.

## Methods

### Study Overview

**Figure 1:**
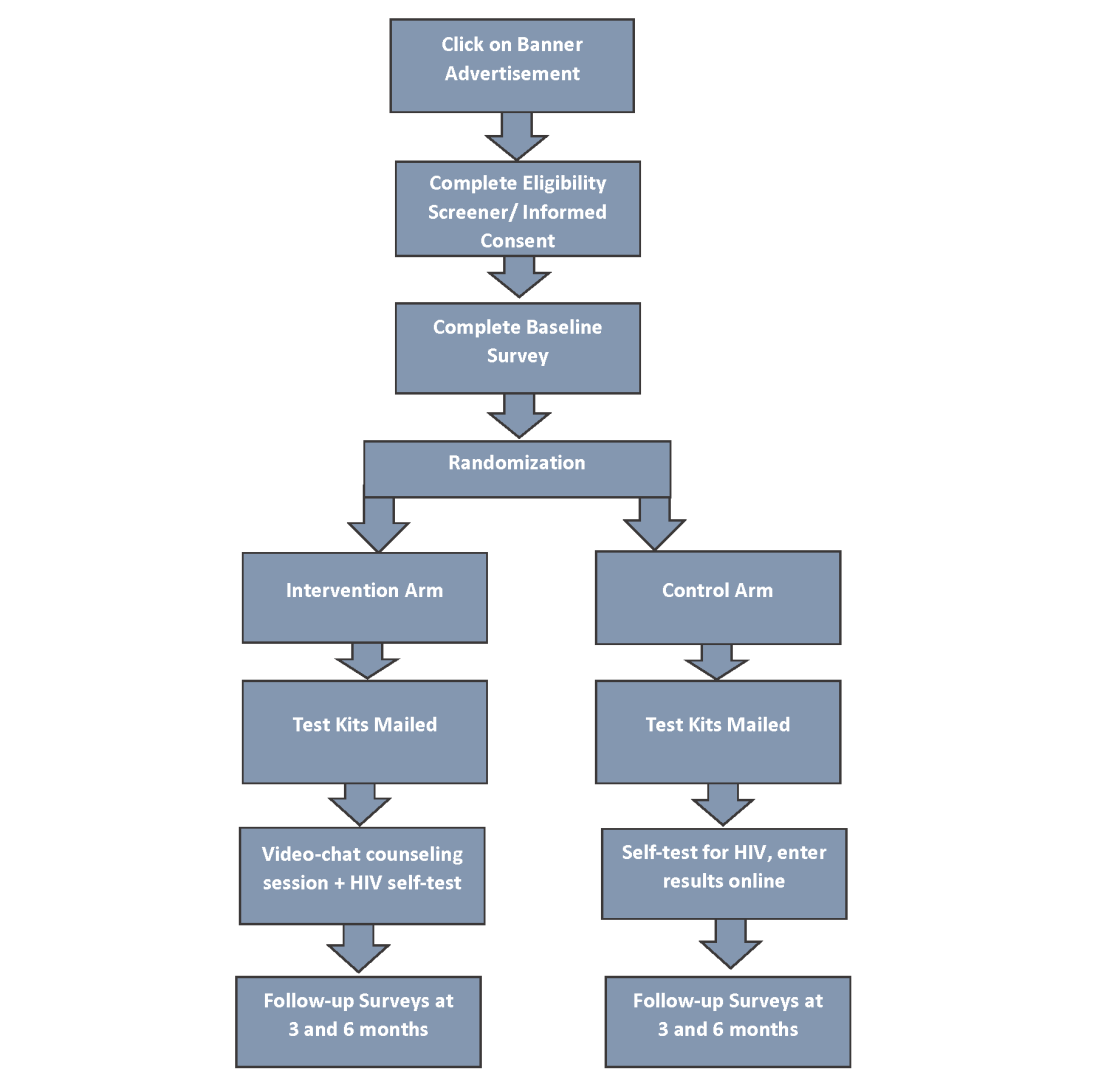
Study flow diagram.

This study is a pilot randomized controlled trial (RCT) of 200 self-identified TY aged 15-24 years, recruited via a range of social media platforms. Participants will be randomized to one of two study arms. TY in the control arm will receive one HIV self-testing kit by mail and will be asked to report their results via a study website. TY in the experimental arm will also receive one HIV self-testing kit via mail, and will conduct this test under the supervision of a remotely-located counselor during a prescheduled video-chat session. Participants in both arms will complete a baseline survey upon recruitment, with repeat surveys at three and six months posttesting (see Figure 1). Participants will receive email and SMS reminders to log into the study website to complete follow-up surveys. The primary outcome for this study is routine HIV testing during the follow-up period. Secondary outcomes include sexual risk-taking behaviors, intervention acceptability, and linkage to care for those who test positive for HIV.

### Participants

Eligible participants will: (1) be aged 15-24 years; (2) have not tested for HIV in the last 12 months; (3) reside in the United States; (4) be willing to have HIV test kits delivered to an address they provide, (5) self-identify as noncis-gender; and (6) have access to a computer, smartphone, or tablet that can support the video-chat software VSee [27], which is an encrypted program compliant with the privacy requirements of the HIPAA.

### Recruitment

**Figure 2:**
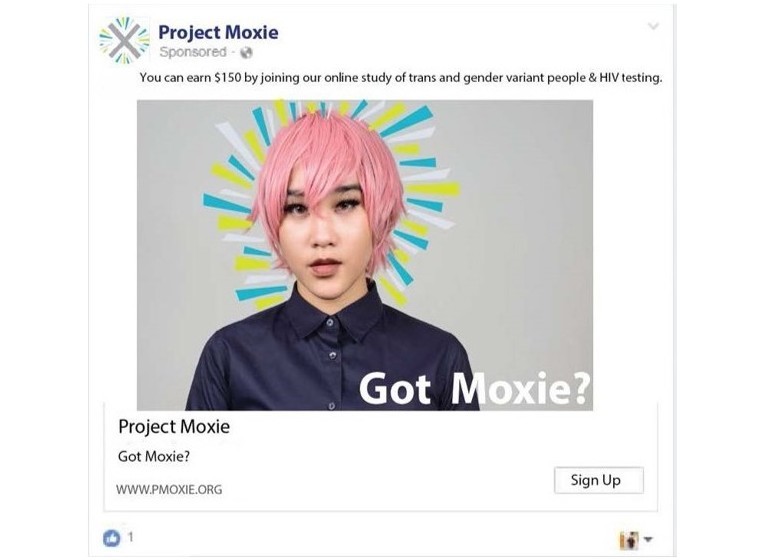
Sample online advertisement.

Participants will be recruited from across the United States via advertisements placed on social media platforms (eg, Facebook, Instagram, and advocacy groups/sites aimed specifically at TY, such as Transgender Alliance and Affirming Transgender Rights; see Figure 2). Online dating apps with transgender users (ie, Scruff) will also be used. Advertisements will include depictions of young people from a variety of racial and ethnic groups to encourage inclusivity and the participation of TY from a variety of backgrounds. Information about the study will also be shared via transgender media personalities’ social media accounts.

When a potential participant clicks on an advertisement, they will be taken to a page containing basic study information, including a short description of study activities. If the individual expresses interest in participating in the study, they will be directed to the study consent form. For participants aged 15-17 years, consent will only be obtained from the participant acting as an emancipated minor. Due to the sensitive nature of the study topic and the stigma and discrimination TY may face from their parents or guardians, requiring parental consent may endanger the safety and wellbeing of the participant. Waiving parental consent allows those who have not fully disclosed their gender identity, or whose guardians are not accepting, to participate in the study.

### Study Procedure

After providing consent electronically, participants will be directed to a short eligibility screener. If eligible, a potential participant will register for the study by providing their contact information. This information includes an email address, mobile phone number, physical mailing address, and preferred choice of name and pronouns. It is important to note that many TY have unstable housing or may not feel comfortable having mail, however discreet, sent to their home address. For this reason, the registration form will ask for a physical address to which the participant feels safe and comfortable receiving mail. As soon as the consent form, screening questionnaire, and site registration are complete, the participant will receive an email with instructions on completing the baseline survey. After completing the baseline survey, each participant will be randomized to either the control arm (HIV self-testing only) or intervention arm (video-based counseling in conjunction with HIV self-testing), using a 1:1 treatment allocation. The treatment assignments will be generated with the use of a pseudo-random number generator, which uses permutated blocks to ensure balance in the number of participants assigned to each arm. The randomization process will generate one of two emails to study participants, indicating whether they will be receiving HIV self-testing or video-based counseling plus HIV self-testing.

#### Control Arm

TY in the control arm will receive one home-based HIV self-testing kit delivered in discreet packaging. In addition to an oral fluid test kit (OraQuick), the package will contain condoms, water-based lubricant packets, and one pair of ear buds. All OraQuick tests will be assigned a specific identification (ID) number that will link to the participant’s study ID number. Upon taking the test, participants will be directed to enter their results on the study website. For those who report a positive result, a message will automatically be sent to the study coordinator. Study staff will then call the participant to offer information regarding places where the participant may acquire a confirmatory HIV test, as well as HIV care services and counseling in their local area or another area of their choosing.

#### Intervention Arm

The intervention arm is a combination of HIV self-testing and HIV test counseling offered remotely via a HIPAA-compliant video-chat session. Participants in the intervention arm also receive one OraQuick test, condoms, lubricant, and earbuds via mail, but will be informed to leave their package *unopened* until directed by the counselor during the video-chat session. Participants will be instructed via email on how to download the VSee software prior to the video-chat session. VSee is available as downloadable software for Windows and Macintosh and as an app in the iOS and Android marketplaces [27]. This software allows participants to log into the video-chat from a desktop, laptop, tablet, or smartphone. Both the software and app versions of VSee contain voice, video, and screen-sharing capabilities to provide a fully-functional counseling session across platforms and devices. VSee provides high-quality video at speeds as low as 50 kilobits per second, allowing full functionality in areas without access to broadband or high-speed cellular data networks [27]. The one-time counseling session lasts approximately 30-45 minutes and consists of two consecutive phases.

Phase one of the counseling session will use elements of motivational interviewing to ascertain reasons (eg, structural, lack of information/misinformation on HIV testing) why the participant has not tested for HIV in the past 12 months. The counselors will also ask about some of the participant’s recent sexual behaviors (eg, number of partners, use of condoms, sexual activities) to establish those that may pose a risk for acquiring HIV. The answers to these questions will form the basis for phase two. Counselors will attempt to provide solutions to mitigate each of the participant’s concerns regarding HIV testing. For structural concerns such as lack of transportation or cost of testing, the counselor might talk about locating local HIV testing services, retailers that sell HIV self-testing kits, and options for free HIV testing in their local area. If the participant reports not testing due to a lack of knowledge of where to test or fear of being recognized at local testing sites, the counselor will assist the participant in finding testing options in nonlocal gender-affirming spaces, and help establish a transportation plan. For participants who report a lack of information or misinformation on the need for HIV testing, the counselor will talk through risk factors for HIV transmission and the CDC recommendations for testing specific to TY. The screen-sharing function in VSee will allow the counselor to share online resources and instruct the participant on their use (eg, how to navigate AIDSVu.com or local health department websites). For those who report fear of stigma or discrimination, the counselor will provide advice on their rights as a patient, including their right to confidentiality, respect, and privacy. The counselor may also share the Gay and Lesbian Medical Association resource list with the participant, which includes *10 Things Transgender Persons Should Discuss with their Health Care Providers* [28]. Through role playing with the counselor, the participant will practice talking about sex and HIV with a health care provider. The counselor will help the participant formulate and practice specific talking points to use with their provider. The counseling will place emphasis on providing the participant with the skills necessary to routinely test for HIV by addressing individual barriers to testing, and giving each participant a supportive and affirming HIV testing experience.

Phase two of the session will consist of testing for HIV. Prior to the test, counselors will offer standard content for risk elicitation and identification of safer sex goal behaviors. The participant, directed by the counselor, will conduct their own test and read their own results. They will then show the counselor their test result for confirmation. Based on the results of the test and the information gathered in phase one, the counselor will assist the participant in developing a prevention or care plan to reduce the risk of acquiring or transmitting HIV (eg, number of sexual partners, frequency of unprotected intercourse). The counselor will describe the behavioral and biomedical interventions appropriate for each participant, such as condom use, partner reduction, decreasing drug or alcohol use, and/or preexposure prophylaxis (PrEP). At the end of the video-chat session, participants who show a positive result will be counseled on the need for timely confirmatory testing and linkage to care. The counselor will arrange a time within one week of the initial session to conduct a second video-chat session. During the second session, the counselor will determine other resources from which the participant may benefit, such as medical case management, mental health care, and/or more comprehensive psychological counseling and services. TY who test positive will also be directly linked to medical care in the second session by connecting them with a provider in their local area or another area designated by the participant. Study staff will follow up on the next business day to ensure that contact was made with the participant’s desired facility. The participant will be contacted at least three more times: (1) to confirm an appointment was scheduled, (2) to confirm that the appointment was attended, and (3) to report confirmatory test results.

#### Counselor Training

Counselors will be trained to use motivational interviewing during a two-day training session. The training will involve didactic presentations on the history, science, and spirit of motivational interviewing, as well as role plays with other counselors acting as study participants. The counseling session protocol was developed with the input of transgender-identified staff members and community members. All counselors will be trained in HIV testing and counseling, and receive specific training on the importance of gender affirmation and working with TY prior to delivering the intervention. Counselors who demonstrate proficiency via audiotaped role plays reviewed by study staff will be cleared to deliver remote counseling to participants. To examine protocol fidelity, all sessions will be audio-taped and research assistants will code and assess a random sample of sessions each month using the *Motivational Interviewing Treatment Integrity* (MITI-4) coding scheme [29-32], and qualitatively assess empathy and motivational interviewing-adherent and nonadherent behaviors. Counselors identified as having treatment drift will receive booster trainings as necessary.

### Measures

Measures will be collected via the baseline, three-month, and six-month online surveys. All measures included in the baseline and follow-up questionnaires were included after a review of the current literature on TY.

#### Outcomes

The primary outcome for this study is routine HIV testing, defined as testing at least once during the follow-up period (ie, every 3-6 months) based on the HIV risk profile of the participant. Secondary outcomes consist of sexual risk-taking behaviors, intervention acceptability at the final survey, and linkage to HIV care (for those who report a positive result at baseline or during the course of the intervention). Key covariates include measures of transphobia and social marginalization.

#### Demographics and HIV Knowledge and Testing History

The demographics section includes measures of age, education, race, ethnicity, sexual orientation, employment, and state of residence. Both the respondent’s sex assigned at birth and current gender identity will be collected. Previous literature on the sexual health of transgender individuals has been criticized for not using discrete categories for trans-masculine/transmale and trans-feminine/transfemale [33]. For this reason, current gender identity will include options for male, female, trans-masculine/male, trans-feminine/female, as well as categories for genderqueer/gender nonconforming, agender/genderfluid individuals, and a participant-driven response option. Items used in previous studies of LGBT persons will measure knowledge regarding the transmission and prevention of HIV [33,34], and knowledge and use of PrEP [34], at baseline and at each follow-up point.

#### HIV Testing

History of HIV testing, including measures of frequency, place of testing, method of testing, and linkage to care (if HIV-positive), will also be collected at baseline. Follow-up surveys will repeat the questions from the baseline and will also ask questions regarding HIV testing since the last survey. These parameters include location of test (eg, home, Acquired Immunodeficiency Syndrome Service Organization, Department of Public Health, or physician), test result, care received (specifically for HIV-positive participants), and reason for testing (ie, routine care versus episodic exposure).

#### Transphobia

Transgender persons face high levels of stigma and discrimination in the United States [12,35-37]. Dimensions of stigma and discrimination among participants will be measured to better understand their associations with testing behaviors among TY. Transphobia will be assessed using subscales from the Gender Minority Stress and Resilience Scale (GMSRS) [38]. Consisting of eight psychometrically-validated subscales, this measure was conceptualized as an assessment of potential facilitators and barriers to engaging in routine HIV testing, and was validated in a sample of 1414 transgender and gender nonconforming persons in the United States.

The *Gender-Related Rejection* and *Gender-Related Victimization* subscales of the GMSRS measure instances of enacted stigma experienced by participants, while the eight-item *Shame* subscale of the *Transgender Identity Scale* [39] will be used to assess internalized transphobia. All three scales use five-point Likert-type questions. Additional measures will also be used to assess the relationship between experiences and/or feelings of transphobia and health outcomes. These measures include the *Affirmation of Gender* scale [38] and the *Self-Admiration* GMSRS subscale [39]. Also using five-point Likert-type response options, the first measures how readily the respondent feels accepted by others in their current gender identity while the second measures the pride one feels in being transgender or gender nonconforming.

#### Social Marginalization

Wilson et al [40] conceptualized transgender social marginalization as having three dimensions: homelessness, incarceration, and participating in commercial sex work. To more fully understand how these dimensions may be associated with routine HIV testing behaviors, participants will be asked about lifetime experiences of homelessness. Participants who have experienced homelessness will also answer whether or not they have been homeless in the past six months. Recent (<12 months) and lifetime history of incarceration, and recent (<3 months) participation in commercial sex work will be assessed by items from the *National Transgender Discrimination Survey* [41]. Commercial sex work is defined in this study as trading sexual activity or favors for food, money, a place to sleep, drugs, or other material goods.

#### Sexual Behaviors

Unprotected sexual intercourse remains the most common route of transmission for HIV in the United States [1]. However, according to the CDC, less than half of transgender men who received an HIV diagnosis in 2016 had any identified or reported sexual risk behaviors [1]. To collect more data on participants’ sexual risk behaviors, behavioral measures adapted from the National HIV Behavioral Surveillance behavioral inventory (and previously used with thousands of MSM by the research team [42,43]) will be used to collect information on sexual behaviors in the past three months. Participants will be asked to estimate the number of anal (and vaginal, if applicable) intercourse partners, as well as condom use or nonuse at each encounter, and the number of times they were the insertive (if applicable) versus receptive partner. The disclosure of HIV status and reported serostatus, or lack thereof, for each partner will also be assessed.

#### Intervention Acceptability and Satisfaction

This is the first study to pair telehealth with HIV self-testing in this population. Understanding how to improve the acceptability and satisfaction of this modality will benefit future studies. TY will report data on the acceptability of the experimental arm at the end of the follow-up period. Two different assessments will be used to measure acceptability: (1) the Self-Evaluation Form (SEF) [44] and (2) the Client Satisfaction Questionnaire (CSQ-8) [45]. The SEF is a brief 13-item questionnaire that elicits information regarding the participant’s experience with the intervention (ie, was the intervention interesting, was it relevant to their life). The CSQ-8 will be used to assess satisfaction with the intervention. The SEF and CSQ-8 will together take approximately 10 minutes to complete. Acceptability of the technology used in the intervention will also be assessed using a survey based on the Unified Theory of Acceptance and Use of Technology , which posits that an individual’s acceptance of a technology is a function of performance, effort, social influence, and facilitating conditions [46]. Technology usage will also be used as a proxy for acceptability, with server logs providing records of user sessions, session length, pages visited, and functions utilized.

#### Linkage to Care

It is important to introduce participants whose self-tests are positive to the HIV treatment cascade and continue following-up on their HIV testing frequency, sexual risk behaviors, and experiences with HIV stigma and transphobia. Per the recent recommendations of the Institute of Medicine [47], indicators of linkage to care will include: (1) attending at least one clinical care appointment, (2) having at least one CD4 test performed, and (3) having at least one viral load test performed within three months of HIV diagnosis [32,48].

### Statistical Analysis

The primary outcome will be the proportion of TY who tested for HIV at least once in the six-month follow-up period. Descriptive statistics of participant characteristics will be presented for all participants and also by study arm. These results will be compared using student t-tests or Wilcoxon rank sum tests for continuous variables, and Chi-square tests for categorical variables. The analysis for each outcome measure is described in detail below.

#### HIV Testing

Analyses will be conducted using logistic regression. The proportion of participants who obtain at least one HIV test within the follow-up period will be calculated for each study arm, along with corresponding 95% confidence intervals. First, the regression model will be fit using study arm only. A second model will control for baseline characteristics, as well as self-reported stigma and discrimination to measure their roles in mediating the outcome.

#### Sexual Risk-Taking

Sexual risk behavior will be defined as any unprotected anal or vaginal intercourse that occurs during the follow-up period with a person who is known by the participant to be HIV-positive, or has an unknown serostatus. The incidence of at-risk sex acts will be calculated as an incidence density, with the numerator being number of individual at-risk sex acts, and the denominator being person-years of follow-up time. Comparisons of the incidence of unprotected sex will be made by comparing incidence densities across arms. Incidence rates per person-year of follow-up will be estimated and compared using methods based on the Poisson distribution and using the generalized estimating equation (GEE) approach. Key covariates include demographic characteristics, previous HIV testing history, and reported stigmas and transphobia. Period incidence rates (three-month incidence density rates) of at-risk sex will be estimated by performing a GEE Poisson regression analysis of the trimonthly counts, implemented using SAS PROC GENMOD [49]. GEE models will control for demographic characteristics, baseline HIV testing history, and stigma, and will also be used to examine interactions between forms of stigma (HIV and transphobia) and sexual risk-taking.

#### Linkage to Care

The percentage of newly-identified HIV-positive participants who attend a comprehensive HIV care visit with a health care provider within three months of diagnosis will be tested for significance across the two study arms using logistic regression analysis. Demographic characteristics and self-reported stigma and discrimination will be controlled in these analyses.

### Incentives

All participants will receive US $30 for completion of the baseline survey and an additional US $30 after completing each of the follow-up surveys. Incentives will be given via email in the form of gift cards to an online retailer, such as Amazon.

### Sample Size

The research team proposes to enroll and maintain a sample of 200 TY aged 15-24 years. In order to achieve this target, recruitment efforts will continue until approximately 250 TY are enrolled. Allowing for 20% loss to follow-up, this approach will produce a sample of 200 TY who are expected to complete the prospective pilot RCT. As a comparison, an ongoing RCT of couples’ HIV counseling and testing in MSM [44] has a retention rate of 90-95%, making a 20% loss-to-follow-up a generous allowance. The sample size is calculated based on the detection of significant changes in each of the outcomes between the control and intervention groups. If data are pooled across participants’ gender identities, the study will have 81%, 92%, and 99% statistical power to observe differences of 10%, 12%, and 15% in the primary outcomes, respectively.

### Trial Registration, Ethics, Consent, and Institutional Board Approval

The Institutional Review Board of the University of Michigan has approved this study (HUM00102906). The study has also been registered on ClincalTrials.gov (NCT03185975). The samples will be reflective of the racial and ethnic diversity of the United States and are deemed to pose no more than minimal risk to the participants.

## Results

Project Moxie began recruitment on June 19, 2017 and has enrolled 130 participants as of August 21, 2017. Self-testing kits have been mailed to a total of 71 participants. Video-chat sessions for 12 participants randomized to the experimental arm have been completed and 58 participants from the control arm have entered their test results into the study database.

## Discussion

Telehealth-based HIV counseling provides TY the opportunity to test for HIV in a comfortable and safe space, as well as problem-solve issues related to testing and risk behavior that are specific to their life experiences. This approach also provides a point of care to TY residing in areas where gender-affirming health care may be scarce, and provides a space free of the discrimination and harassment TY often encounter while trying to access health care resources.

A potential limitation is the protocol’s reliance on existing health care resources for linkage to care and follow-up. TY may participate in Project Moxie due to a lack of locally-available resources that are accepting and gender-affirming. However, after their initial session, those who test positive and/or require additional services may find limited options in their local area and may have difficulty entering into the continuum of care. The current price of self-test kits (approximately US $30-40) may be prohibitive for many TY and could be a barrier to routine testing in areas without gender-affirming or subsidized HIV testing services.

Despite these limitations, pairing HIV testing with counseling and linkage to care is an innovative approach to increasing access to sexual health care. Using telehealth to link high-risk TY to HIV testing and care has the potential to reduce the high rate of HIV and STI transmission among this high-risk and often-marginalized population.

## Abbreviations

CDC: Centers for Disease Control and Prevention
CSQ-8: Client Satisfaction Questionnaire
GEE: generalized estimating equation
GMSRS: Gender Minority Stress and Resilience Scale
HIPAA: Health Information Portability and Accountability Act
HIV: human immunodeficiency virus
ID: identification
LGBT: lesbian, gay, bisexual, transgender
MSM: men who have sex with men
PrEP: preexposure prophylaxis
RCT: randomized controlled trial
SEF: Self-Evaluation Form
SMS: short message service
STI: sexually transmitted infection
TY: transgender and gender nonconforming youth
